# Delay in Diagnosis of Adrenal Insufficiency Is a Frequent Cause of Adrenal Crisis

**DOI:** 10.1155/2013/482370

**Published:** 2013-06-24

**Authors:** Lucyna Papierska, Michał Rabijewski

**Affiliations:** ^1^Clinic of Endocrinology, The Centre of Postgraduate Medical Education, Warsaw, Poland; ^2^Department of Internal Diseases, Diabetology and Endocrinology, Medical University of Warsaw, Poland

## Abstract

Delay of diagnosis of primary adrenal insufficiency (PAI) leads to adrenal crisis which is potentially lethal complication. The objective of our work was an assessment whether the establishment of diagnosis of adrenocortical insufficiency in Poland is so much delayed as assessed in the past. We have analysed data from 60 patients with diagnosis of PAI established in our department during the past 12 years and who are still under our care. We found that the time to diagnosis of primary adrenal insufficiency in Poland exceeds 3 months in every patient and 6 months in patients admitted with symptoms of adrenal crisis. Forty-four percent of patients were diagnosed only just after the hospitalisation due to crisis, despite the evident signs and symptoms of PAI. Lack of appetite and loss of body weight occurred in all patients and for that reason a diagnosis of chronic gastric and duodenal ulcer disease was the most often incorrect diagnosis. After the proper diagnosis and treatment, in the course of 1–11 years of observation, there was only 6 imminent adrenal crises in 5 patients. Our results indicated that training of primary care physicians in the field of recognising and treatment of adrenal insufficiency is still essential.

## 1. Introduction 

The primary adrenocortical insufficiency, that is, Addison's disease, has already been identified and described in the mid-19th century [1]. Adrenal tuberculosis was the most common cause of primary adrenocortical insufficiency for more than a century. However, autoimmune adrenocortical insufficiency is the predominating form in the last twenty years, which occurs in 93–140 million of various populations, and new cases amount to about 5/million yearly [2, 3]. On the grounds of the foregoing data one can presume that primary adrenocortical insufficiency affects at least 4000 persons in Poland, and we can expect to encounter about 200 new diagnoses of this disease yearly [4]. 

An undiagnosed and untreated primary adrenocortical insufficiency is a lethal disease. According to Zelissen, its most dangerous complication, that is, adrenal crisis, occurs before Addison's disease is diagnosed in half of the patients. History-taking after adrenal crisis cure revealed that first symptoms occurred in every second patient already at least one year before hospitalization [5]. Bleicken et al. stated that 50% of affected individuals report evident symptoms of adrenocortical insufficiency at least half a year prior to establishment of the diagnosis, and every fifth patient waits for a correct diagnosis for more than 5 years [6]. In Norwegian study every third patient was diagnosed very quickly—already after one month of the symptoms, but 40% of affected persons waited for diagnosis more than 6 months [7]. The symptoms of adrenal insufficiency are not specific, suggesting an gastrointestinal disease (abdominal pains, lack of appetite, nausea and vomiting, body mass loss), primary muscle disease (muscular pains and myasthenia) or depression (weakness, mood depression, and hypothymia). The arterial blood pressure decreases in most cases, but there can be present only orthostatic hypotension [8, 9]. The unspecificness of the symptoms can delay establishment of the diagnosis, which increases the risk of the potentially lethal complication, that is, adrenal crisis [10]. The adrenal crisis is a catecholamine-resistant shock that results from cortisol deficiency. It is accompanied by electrolyte disturbances distinctive of adrenocortical insufficiency in the form of hyponatremia and hyperkalemia, sometimes also by hypoglycemia and hypercalcemia. An imminent adrenal crisis is a period of intensified symptoms of adrenocortical insufficiency in the form of nausea and vomiting, substantial hypotension, and severe debilitation precluding daily activities. If a treatment with hydrocortisone is not started immediately, these symptoms will intensify and turn into a crisis. 

During the last years, several actions, for example, the EU-funded “Euradrenal” project, were undertaken to improve doctors' knowledge of diagnosis and treatment of adrenocortical insufficiency and adrenal crisis. These topics are dedicated not only to endocrinologist but also to internist and family doctor trainings as well as first-aid service trainings. Furthermore, two new Polish-language textbooks of endocrinology were published that contain voluminous chapters concerning adrenal gland diseases. Patients use numerous websites, which allow them to search for potential causes of distressing symptoms or ask for help from other patients or sometimes even doctors. 

The objective of our study was an assessment whether the establishment of diagnosis of adrenocortical insufficiency in Poland is so much delayed as assessed in the papers cited above and a determination of the number of patients in whom adrenal crisis is the first symptom of this disease. 

## 2. Material and Methods

The material consisted of 60 patients (48 women and 12 men aged 20–70 years) in whom primary adrenocortical insufficiency was diagnosed for the first time in our department in 2000–2012 and who are still under our care. The profile of symptoms preceding the establishment of diagnosis was analysed based on anamnesis. Each patient answered the standard questionnaire used in our hospital. The patient's history was taken and the physical examination was performed by one of two investigators. Based on collected data, the time from the beginning of ailments to diagnosis was determined. The concentration of ACTH and cortisol at admission, functional state of thyroid gland, and presence of antiadrenal and antithyroid antibodies were determined. Cortisol concentrations and antibodies titters were assessed by a chemiluminometric assays. ACTH levels were measured by a radioimmunometric assay. These parameters in groups with or without adrenal crisis were compared using Student's *t*-test. 

## 3. Results 

All the diagnoses were confirmed by a low blood concentration of cortisol in the morning (89.6 nmol/L ± 22.4; normal range 140–799 nmol/L), a high concentration of ACTH (184.8 pmol/L ± 78.3; normal range: 2.2−11 pmol/L). An additional ^1−24^ACTH (250 mcg) stimulation test was carried out in 16 patients, which did not show a significant increase in cortisolemia in the 30th and 60th test minute.

In the analysed group of patients, the diagnosis of adrenocortical insufficiency was for the first time established during the adrenal crisis in 13 patients (22%) and in other 13 (22%) patients just as symptoms of imminent adrenal crisis appeared. Five of the patients with symptoms of imminent adrenal crisis were hospitalized in the other wards during the last year before admission to our hospital. They were admitted with the same symptoms, treated symptomatically (infusions of normal saline, antiemetic drugs, proton pump inhibitors, and antibiotic therapy), and discharged home without establishment of diagnosis. 

In the groups with adrenal crisis or imminent adrenal crisis, only three patients (11%) were treated with endocrinological procedures in the past—1 under continuing endocrinological care for hypothyroidism and 2 for hyperthyroidism in the course of Graves' disease in the past (1 and 30 years prior to diagnosis of adrenocortical insufficiency). In the group without adrenal crisis, 18 patients (53%) suffered from an autoimmune disease that was a part of type 2 autoimmune polyglandular syndrome (APS-2): 10 patients had hypothyroidism in the course of Hashimotos' thyroiditis, 5 hyperthyroidism in the course of Graves' disease, 3 megaloblastic anemia, and 2 premature ovarian failure. Vitiligo occurred in 3 patients in the group with adrenal crisis and in 6 patients in the group without adrenal crisis. In total, elements of the APS-2 were found in 6 (23%) patients with adrenal crisis and in 24 (71%) patients without adrenal crisis before diagnosing adrenal insufficiency. Two patients (7%) in the group with adrenal crisis and 16 patients (47%) in the group without adrenal crisis were under care in endocrinology clinics in the last year before the diagnosis of adrenocortical insufficiency was established, but none of them had evaluation of adrenal function at that time. 

The titre of antibodies against 21-hydroxylase was significant in 49 (82%) patients: 11 (85%) with imminent crisis, 10 (78%) with crisis, and 28 (82%) without crisis. Metastatic and infiltrative changes in the adrenal glands were excluded by abdominal ultrasonography, and additional CTs of abdomen were performed in patients with negative antibody titres. In all examined patients CT scans revealed small adrenal glands, without signs suggesting tuberculosis, infiltrative, or metastatic diseases. Moreover we excluded 21-hydroxylase deficiency in these patients (the levels of 17 OH-progesterone were below 1.0 ng/mL). 

Positive titres of antithyroid antibodies (aTPO, aTG) were found in 26 patients (43%), and in this group 11 patients already had an hypothyroidism treated with thyroxine at the dose of 50–125 mcg. On the grounds of raised TSH concentrations (6.2 ± 1.8), a thyroxine dose increase was also recommended for 7 patients. An excessive (double) thyroxine dose increase was the cause of adrenal crisis in one female patient in the abovementioned group. During further observation at an outpatient clinic, a positive titre of antithyroid antibodies was found in 5 more patients, and 7 patients with positive antibody titres have developed a hypothyroidism. Eleven patients suffered from hyperthyroidism in the course of Graves' disease in the past (1–31 years prior to the establishment of diagnosis of adrenocortical insufficiency), and an adrenocortical insufficiency was diagnosed during the treatment of hyperthyroidism in 4 patients. Two patients in this group being in a severe state have been hospitalized (1 patient with adrenal crisis and 1 patient with imminent adrenal crisis and fully symptomatic hyperthyroidism). 

Five patients suffered from type 1 diabetes before the diagnosis of adrenocortical insufficiency. A few months before the diagnosis of Addison's disease, it was necessary to decrease insulin doses in all these patients because of recurrent hypoglycemia (most often at night and in the early morning). In this group, 1 patient having adrenal crisis with hypoglycemia has been hospitalized. 

Lack of appetite, nausea, wasting, orthostatic hypotension, and hyperpigmentation of the skin have been recognized to be evident signs and symptoms of adrenocortical insufficiency. In the group of patients with adrenal crisis at admission, the mean time from appearance of the abovementioned signs to the diagnosis was 9.1 ± 3.5 months; the diagnosis was established significantly later in comparison with the patients without adrenal crisis (5.8 ± 2.8 months; *P* = 0.05). When comparing the patient subgroup that had other concomitant autoimmune diseases and was attended by an endocrinology clinic with the subgroup without autoimmune diseases, it was demonstrated that the time until diagnosis was significantly shorter in the group with other elements of the APS (4.6 ± 3.7 months versus 11.3 ± 9.0 months; *P* = 0.007). 

Lack of appetite and wasting occurred in all the subjects and usually were the first symptoms of disease. The mean body mass loss was greater in the group with adrenal crisis (10.2 ± 3.5 kg) compared with the group without adrenal crisis (7.0 ± 2.8 kg; *P* = 0.01). For these symptoms diagnostics, a gastroscopy was performed in 20 patients (33%), and 27 patients (45%) with diagnosed or suspected gastritis received a chronic treatment with proton pump inhibitors. 

Skin hyperpigmentation was found in 57 of 60 patients. Skin darkening occurred in the palmar creases, elbow flexures, and areolas of the mammas in all the patients and affected the whole skin in 50 patients. This sign did not occur only in 3 patients due to (already diagnosed) generalized vitiligo.

An orthostatic hypotension (defined as the decrease in diastolic and systolic arterial pressure by at least 10 mm Hg) was found in all the patients in the group without adrenal crisis. According to the definition, patients with adrenal crisis or imminent adrenal crisis had a substantial hypotension or shock. It was impossible to collect data on arterial blood pressure values during the last year preceding the diagnosis of adrenocortical insufficiency because arterial pressure was not measured in two positions at subsequent visits, and sometimes this parameter was not assessed at all.

Muscular pains affected 22 (37%) patients, which have been mostly associated with exercise or infection by the patients. These pains occurred mainly in the muscles of the back and lower limbs and disappeared after rest. All the patients reported decreased muscle power, general weakness, and fatigability. 

Hyponatremia (sodium concentration below 137 mEq/L: 130.7 ± 4.01 mEq/L) occurred in all the patients hospitalized for adrenal crisis or imminent adrenal crisis, and sodium concentrations were decreased or at lower normal range (134.5 ± 3.7 mEq/L) in the group without adrenal crisis. Hyponatremia was significantly more severe (*P* = 0.006) in the group with adrenal crisis. It occurred at least three months before the establishment of the diagnosis in 34 patients (57%), but this was not followed by a correct diagnosis at that time. 

Hyperkalemia (potassium concentration above 5.1 mEq/L) was found in 11 patients (85%) hospitalized for adrenal crisis and in 7 patients (54%) with imminent adrenal crisis. Hyperkalemia was not found in the group without adrenal crisis. The mean potassium concentration was 4.9 ± 0.4 mEq/L in the group with adrenal crisis and 4.35 ± 0.3 mEq/L in the group without adrenal crisis (*P* = 0.001). 

The comparison between two groups of patients is shown in Table 1. 

After establishing the diagnosis in all the patients, a treatment with 15–30 mg hydrocortisone per day in divided doses and 25–100 mcg fludrocortisone per day was started. The patients are attended by the Endocrinological Clinic, and the observation time is 1–11 years. During this period of observation, 6 imminent adrenal crises occurred in 5 patients. Three of these adrenal crises (2 in one patient) were caused by hydrocortisone absorption disturbances in the course of peptic ulcer or acute gastritis, and another one followed a traffic accident injury after which the initially increased glucocorticoid dose has been decreased too fast, and the other ones were caused by insufficient dose increases during a febrile disease. 

## 4. Discussion

The treatment of primary adrenal insufficiency is simple. It consists mainly in supplementing the deficient glucocorticoids and mineralocorticoids, which brings about a prompt and spectacular improvement [11, 12]. However, the lack of the proper diagnosis of adrenal insufficiency can have tragic sequel since this pathology leads to adrenal crisis and, if not treated with parenterally administered glucocorticoids, is lethal. Final diagnostics require a few simple determinations—a blood cortisol and ACTH concentration measurement and a determination of cortisol metabolites excretion in 24-hour urine. The tetracosactide (^1-24^ACTH) stimulation test is a dynamic test characterized by high sensitivity in the diagnostics for primary adrenocortical insufficiency in which an increase in cortisolemia after ACTH analogue administration is not found [13, 14]. 

Ruling out or confirmation of suspected primary adrenocortical insufficiency is usually a simple task, but a diagnostics focus on this disease is still delayed considerably. The cited above papers demonstrate that symptoms lasted for more than one year in every fourth patient and in many cases even for more than 5 years [6]. We did not find in our material such a substantial delay in establishing the diagnosis; nevertheless, the symptoms were present during at least the last six months before the admission to hospital of patients in a severe state from adrenal crisis. The shorter duration of symptoms reported by our patients in comparison with the Bleicken's paper can be a result of the differences in the methodology of taking the anamnesis. The duration of symptoms was reported by our patients at the first visit in outpatient clinics or at admission to hospital. Data were collected retrospectively in the cited Bleicken's study, so the patients had more time for an analysis of symptoms that occurred in the past (sometimes this could lead to an incorrect attribution of symptoms of other diseases to adrenocortical insufficiency). Furthermore, in the analysis of symptom duration, we did not include the least specific symptoms, that is, general weakness and fatigability, since a large percentage of our patients have other diseases and the worsening of physical efficiency in the past could be a result of hypothyroidism, anaemia, or vitamin B12 deficiency accompanying Addison's disease. The third reason why the diagnosis was established relatively promptly compared with the group described by Bleicken is that unlike the cited study our paper concerned only primary adrenocortical insufficiency, which presents with more pronounced symptoms and the very distinctive darkened skin (according to the Williams Textbook of Endocrinology [9] in 94% of patients and in our material in 95% of patients). The establishment of diagnosis in our patients was also easier because some patients (32%) were regularly attended by endocrinology outpatient clinics for other autoimmune diseases. In this subgroup of patients, the time until diagnosis was indeed significantly shorter compared with patients untreated with endocrinological procedures so far. However, the treatment with thyroxine could intensify symptoms of adrenocortical insufficiency in patients with hypothyroidism due to the increased cortisol clearance through thyroxine. In our opinion, a drastic thyroxine dose increase brought about an adrenal crisis in one female patient. Endocrinologists should remember that increased TSH level may result from cortisol deficiency as well as from hypothyroidism. 

The analysis of adrenal insufficiency symptoms showed that a lack of appetite resulting in wasting was always present in our patients and usually was the first symptom of disease. Thus, a diagnosis of chronic gastric and duodenal ulcer disease was the most often incorrect diagnosis. Hypothyroidism, depression, anorexia nervosa, fibromyalgia, “neoplastic disease of unknown primary site,” collagenosis, myocarditis, and also chronic fatigue syndrome were the other established diagnoses in few patients. Muscular pains occurred in every third patient. According to the Williams Textbook of Endocrinology [9], these pains occur even rarer and affect only 13% of patients, whereas 36% of patients with primary insufficiency reported such pains in the Bleicken's material [6]. Only few persons in our series reported salt craving, so we did not include this symptom in our analysis though it is recognized to be characteristic by most authors. During the last weeks before hospitalization, the symptoms were very intensive in all the patients with adrenal crisis or imminent adrenal crisis at admission. They precluded daily activities and were the reason for temporary work disability. However, this has not resulted in an intensification of diagnostic procedures. Another distressing fact is that no discriminating diagnostic procedures for the severe hyponatremia were performed, but only a symptomatic treatment was applied in 5 patients who were previously hospitalized during an intensification of symptoms. Moreover, the assessment of blood sodium concentration is not a standard in primary care in Poland (contrary to blood kalium examination). Hyponatremia was present in all our patients hospitalized for adrenal crisis or imminent adrenal crisis. Sodium concentrations were decreased or at lower normal range even in the group without adrenal crisis. It should draw attention to the importance of examination of natremia in patients with hypotony, weakness, loss of appetite, and wasting. On the other hand, in the patients with recognized hyponatremia, assessment of adrenal function must be performed (at least morning cortisol blood level measurement). 

## 5. Conclusion

In conclusion, it should be stated that a delay in performing diagnostics for primary adrenocortical insufficiency still existed during the last twelve years though it is not so extreme as described in the older papers. The unspecificness of symptoms brings about several incorrect diagnoses, mostly diagnoses of upper gastrointestinal tract disease. As a result of the delay in establishing diagnoses, a large percentage of patients become hospitalized for adrenal crisis. Primary care physicians still require regular training in the establishment of diagnosis of adrenocortical insufficiency with special focus on signs, symptoms, and electrolyte disturbances most distinctive of this disease. 

## Figures and Tables

**Table 1:** Comparison of two groups: with and without crisis at the time of adrenal insufficiency diagnosis.

	Without crisis	With crisis or imminent crisis	*P*
Number of patients	34	26	
Age at diagnosis (years)	39.2 ± 14.7	39.0 ± 13.7	ns
F/M ratio (*n*/female %)	28/6—82% f	20/6—78% f	ns
Morning cortisol levels (nmol/L)	116.4 ± 36.9	54.5 ± 26.0	0.001
Mean levels of ACTH (pmol/L)	149.1 ± 101.3	220.4 ± 136.4	ns (0.06)
Coexistent elements of aps II (%)	71%	23%	0.01
Treatment by endocrinologist in the past (%)	47%	7%	0.01
Time to diagnosis (months)	5.8 ± 2.8	9.1 ± 3.5	0.05
Body mass loss (kg)	7.0 ± 2.8	10.2 ± 3.5	0.01
Blood sodium concentration (mEq/L)	134.5 ± 3.7	130.7 ± 4.01	0.006
Blood potassium concentration (mEq/L)	4.35 ± 0.3	4.9 ± 0.4	0.001
Positive 21-OHAbs titers (%)	82%	81%	ns
